# Copy Number Variations Burden on miRNA Genes Reveals Layers of Complexities Involved in the Regulation of Pathways and Phenotypic Expression

**DOI:** 10.1371/journal.pone.0090391

**Published:** 2014-02-28

**Authors:** Avinash M. Veerappa, Megha Murthy N, Sangeetha Vishweswaraiah, Kusuma Lingaiah, Raviraj V. Suresh, Somanna Ajjamada Nachappa, Nelchi Prashali, Sangeetha Nuggehalli Yadav, Manjula Arsikere Srikanta, Dinesh S. Manjegowda, Keshava B. Seshachalam, Nallur B. Ramachandra

**Affiliations:** 1 Genetics and Genomics Lab, Department of Studies in Zoology, University of Mysore, Manasagangotri, Mysore, Karnataka, India; 2 Department of Anatomy, Yenepoya Medical College, Yenepoya University, Mangalore, Karnataka, India; 3 Department of Neurology, JSS Hospital, Mysore, Karnataka, India; 4 Nitte University Centre for Science Education & Research, K S Hegde Medical Academy, Nitte University, Deralakatte, Mangalore, Karnataka, India; Tel Aviv University, Israel

## Abstract

MicroRNAs are involved in post-transcriptional down-regulation of gene expression. Variations in miRNA genes can severely affect downstream-regulated genes and their pathways. However, population-specific burden of CNVs on miRNA genes and the complexities created towards the phenotype is not known. From a total of 44109 CNVs investigated from 1715 individuals across 12 populations using high-throughput arrays, 4007 miRNA-CNVs (∼9%) consisting 6542 (∼5%) miRNA genes with a total of 333 (∼5%) singleton miRNA genes were identified. We found miRNA-CNVs across the genomes of individuals showing multiple hits in many targets, co-regulated under the same pathway. This study proposes four mechanisms unraveling the many complexities in miRNA genes, targets and co-regulated miRNA genes towards establishment of phenotypic diversity.

## Introduction

Copy Number Variations (CNVs) are a segment of DNA of >1 kb with a variable copy number compared with the reference genome [1]. CNVs are a widespread and common phenomenon among humans [2], [3] and no single human without CNVs has yet been identified. CNV occurrence can be limited to a single gene or a contiguous set of genes and results in dosage-sensitiveness, which may contribute significantly towards human phenotypic variability, complex behavioral traits and disease susceptibility. CNVs in one or more genes can cause functional loss, either by disrupting a regulatory element, generating novel fusion products, or through position effect, with various possible outcomes, including imprinting and differential allelic expression [4].

CNVs are prevalent in both coding and non-coding regions of the genome encompassing both genes and regulatory regions. These coding regions also contain MicroRNAs (miRNA) genes across all chromosomes and makes up about 4% of the coding genes. miRNAs are ∼22 base small RNA sequences, derived from the pre-transcript of ∼70–120 bases that affect significant biological processes by repressing the translation of coding genes by binding to specific sequence in the 3′UTR of target genes [5]. These miRNA genes regulate the production of protein for >30% of all human coding genes [6]. Over expression of certain miRNA genes is found to contribute towards cancer and other diseases [7], and some miRNAs have multiple mRNA targets, and some of these targets are also regulated by multiple miRNAs [8]. miRNAs are involved in crucial biological processes, including development, differentiation, apoptosis and proliferation, through imperfect pairing with target mRNAs of protein-coding genes and the transcriptional or post-transcriptional regulation of their expression [9].

CNVs in miRNA genes affect binding and regulation of miRNA target genes [10]. CNV-miRNAs are involved in many processes and diseases, including organ development, angiogenesis, and male fertility. CNV-miRNAs are known to form regulatory loop for several transcripts and downstream target genes and the miRNAs present in deleted and duplicated CNV loci alter the dosage of the crucial genes by impairing the processing miRNAs by targeting key molecules [11]. Previous CNV-miRNA studies have been performed on HapMap genomes [9], [12] without providing population specific CNV-miRNA distribution and only reasoned the genotypic complexities contributed by the CNVs on miRNA subjectively.

miRNAs act by modulating gene expression by inhibiting the translation and facilitating the degradation of their targeted mRNAs, and these are found aberrantly expressed in many diseases including cancer, and such studies have concentrated only on the genotypic complexities involving either miRNA genes or its target genes, and have failed to collectively analyze the complexities in the individual genomes [13]–[15]. In the present investigation, we have made an attempt to identify the CNV burden on miRNA genes across 12 populations which report the first drafts of population-specific CNV-miRNA maps and provide several layers of genotypic complexities and phenotypic expressions.

## Materials and Methods

For this study, a total of 1715 individuals involving 43 normal members from randomly selected twelve families residing in Karnataka, India with different age group members ranging from 13–73 years, 270 HapMap samples covering CEU (CEPH collection), CHB (Han Chinese in Beijing, China), JPT (Japanese in Tokyo, Japan) and YRI (Yoruba in Ibadan, Nigeria) populations, 31 Tibetan samples, 155 Chinese samples, 472 of Ashkenazi Jews replicate 1, 480 of Ashkenazi Jews replicate 2, 204 individuals from Taiwan, 55 from Australia and 64 from New World population (Totonacs and Bolivians), were selected for the CNV analysis in the genome. The 270 individuals sample data from the four populations was obtained from the International HapMap Consortium [16]. The samples for the HapMap come from a total of 270 people: the 30 both-parent-and-adult-child trios from the Yoruba people in Ibadan, Nigeria, 45 unrelated Japanese individuals in Tokyo, 45 unrelated individuals Han Chinese in Beijing, and the 30 both-parent-and-adult-child trios from CEPH. The raw, unprocessed data from Affymetrix Genome Wide SNP 6.0 array for the 31 individuals of Tibet population was obtained from the ArrayExpress Archive at the European Bioinformatics Institute to identify regions of the genome that have undergone positive selection in a high-altitude Tibetan population. The following datasets were obtained from the ArrayExpress archive with the accession numbers E-GEOD-21661, E-GEOD-29851, E-GEOD-30481, E-GEOD-15826, E-GEOD-23636, E-GEOD-23201, E-GEOD-33355 and E-GEOD-33356. Data has been made publicly accessible through the University of Mysore Genome Centre Database (URL: http://umgc.uni-mysore.ac.in/index.php/search/cnv).

5ml EDTA blood was collected from each member of the Indian study group and genomic DNA was extracted using Promega Wizard Genomic DNA purification kit. The isolated DNA was quantified by Bio-photometer and gel electrophoresis. This research was approved by the University of Mysore Institutional Human Ethics review committee (IHEC). Written informed consent was obtained from all sample donors and the IHEC approved the sample consent procedure. Written informed consent was obtained from parents/guardians in the cases of participants being minors.

### Genotyping

Genome-wide genotyping was performed using an Affymetrix Genome-wide Human SNP Array 6.0 chip and Affymetrix CytoScan High-Density (HD) Array having 1.8 million and 2.6 million combined SNP and CNV markers with the median inter- marker distance of 500–600 bases. These chips provide maximum panel power and the highest physical coverage of the genome [17]. Genotyping quality was assessed using Affymetrix Genotyping Console Software. Copy Number Analysis Method offers two types of segmenting methods, univariate and multivariate. These methods are based on the same algorithm, but use different criteria for determining cut-points denoting CNV boundaries.

### Algorithms for Copy Number state calling

#### BirdSuite (v2)

BirdSuite [18] is a suite originally developed to detect known common CNPs based on prior knowledge, as well as to discover rare CNVs, from Affymetrix SNP 6.0 array data. To do this, it incorporates two main methods; the “Birdsuite” algorithms and the“Canary” [19]. The Birdsuite algorithm uses a Hidden Markov model (HMM) approach to find regions of variable copy number in a sample. For the HMM, the hidden state is the true copy number of the individual's genome and the observed states are the normalized intensity measurements of each array probe. CNV calls from the Canary and Birdsuite algorithms were collated for each sample, and kept as long as they met the following criteria: i) Birdsuite calls with a log10 of odds (LOD) score (Odds Ratio) greater than or equal to 10 (corresponding to an approximate False Discovery Rate of ∼5%), ii) Birdsuite calls with copy number states other than 2 were retained; iii) Canary CNP calls with CN states different from the population mode were retained.

#### CNVFinder

CNVFinder developed at the Welcome Trust Sanger Institute uses a dynamic, multiple-threshold based approach to allow robust classification of copy number changes in data of varying qualities. This algorithm makes two main assumptions i) that the majority of data points are normally distributed around a log2 ratio of zero, and ii) that data points falling outside of the centralized log2 ratio distribution are representative of a difference in copy number between test and reference genome.

#### Canary

CNP analysis was performed using the Canary algorithm. Canary was developed by the Broad Institute for making copy number state calls in genomic regions with CNPs. Canary algorithm computes a single intensity summary statistic using a subset of manually selected probes within the CNP region. The intensity summaries are compared in aggregate across all samples to intensity summaries previously observed in training data to assign a copy number state call.

### Genotyping Console

After processing CEL files and the Birdseed to call genotypes, we used the Genotyping Console (GTC v.3.0.2) to detect CNVs from the Affymetrix 6.0 array for samples that passed initial QCs. The default parameters of >1 Kb size and >5 probes in this algorithm were used.

### Data Analysis

Genome-wide CNV study was carried out using SVS Golden Helix Ver. 7.2 [20] and Affymetrix Genotyping Console software as prescribed in their manuals [21]–[23]. Eigenstrat method was used to avoid possibility of spurious associations resulting from population stratification. Bonferroni correction was employed for multiple testing and the corrected data were then used for CNV testing. Bonferroni methods for population data genotyped on the Affymetrix 6.0 platform was α = 0.05 thresholds between 1×10^−7^ and 7×10^−8^.

Analyzing the collated data from both BirdSuite and Canary algorithms increased the stringency on those meeting the CNP calls with a log10 of odds score greater than or equal to 10 corresponding to a False Discovery Rate of ∼5%. All SNPs that were called using Birdseed v2algorithm had a Quality Control (QC) call rate of >97% across individuals. All the subjects and members with SNPs that passed SNP QC procedures were entered into the CNV analysis. Filters were set for ID call rates for the overall SNPs to identify IDs with poor quality DNA, if any. The CNV calls were generated using the Canary algorithm. In AGCS, contrast QC has to be >0.4 to be included in the CNV analyses. In this study, contrast QC observed was >2.5 across all samples showing a robust strength. To control for the possibility of spurious or artifact CNVs, we used the EIGENSTRAT approach of Price et al., [24]. This method derives the principal components of the correlations among gene variants and corrects for those correlations in the testing. We removed 55 individuals from the study group because they were extreme outliers on one or more significant EIGENSTRAT axes and further dropped 543 CNVs in the members selected for the study for not meeting the required QC measures. CNVs were considered validated when there was a reciprocal overlap of 50% or greater with the reference set. Though the Jaccard statistic is sensitive to the number of CNVs called by each algorithm (ideally each two algorithms would detect similar number of CNV calls), the relative values between the different comparisons of algorithms/platform/site are very informative. All the overlap analyses performed have handled losses and gains separately except when otherwise stated, and were conducted hierarchically. The calls from the algorithms that were called in both were not considered; instead, they were collated so that the relative values between the different comparisons of algorithms/platform/site are still very informative.

### HD-CNV

In order to compare and identify CNV-miRNA between samples of the same and other population as hotspots and rare, and to also correlate their abeyant effects on a wide variety of biological contexts, HD-CNV [25] (Hotspot Detector for Copy Number Variants), was used to analyze and detect recurrent CNV regions by finding cliques in an interval graph generated from the input. HD-CNV requires CNV calls as an input to detect recurrent regions based on percentage overlap. Here, CNV events are treated as nodes in an interval graph and are used to represent regions (intervals) on a real line, and edges are added where intervals overlap as postulated by Lekkerkerker et al. [26]. Based on this, Butler et al. [25], modified and added edges between nodes that share the base pair overlap required to consider two CNV events part of a merged region (default 40%) and the overlap required for a family (region with highly similar CNV events, default 99%). Merged regions, therefore, contain a collection of CNV events where each overlaps all others in the merged region by the minimum overlap specified, and indicate the genomic location where those groups of overlapping CNVs are found. [25]. The output graph generated by the HD-CNV was then visualized using Gephi graph creation software.

### Generation of CNV map

The shared map of 6542 miRNA genes under CNVs across all chromosomes was generated using the Circos software package [27].

### Breakpoint Validations

To validate some of the identified miRNA-CNV breakpoints from this study, PCR was performed on 400 randomly chosen individuals for 4 miRNA-CNV breakpoints. A chimeric primer flanking normal and deleted/duplicated sequence was designed so as to bind only to the miRNA-CNV breakpoints. Samples that do not contain these specific miRNA-CNVs, would fail to amplify. PCR amplification was performed at 95°C for 3′ followed by 35 cycles of 94°C for 30′′, 62°–68°C for 30′′, and 72°C for 5′ in Kyratec PCR System (Kyratec, Australia). All PCR products were analyzed on 1% agarose gels and documented with a Vilber Lourmat Imaging system (Vilber Lourmat, France).

## Results

**Figure 1:**
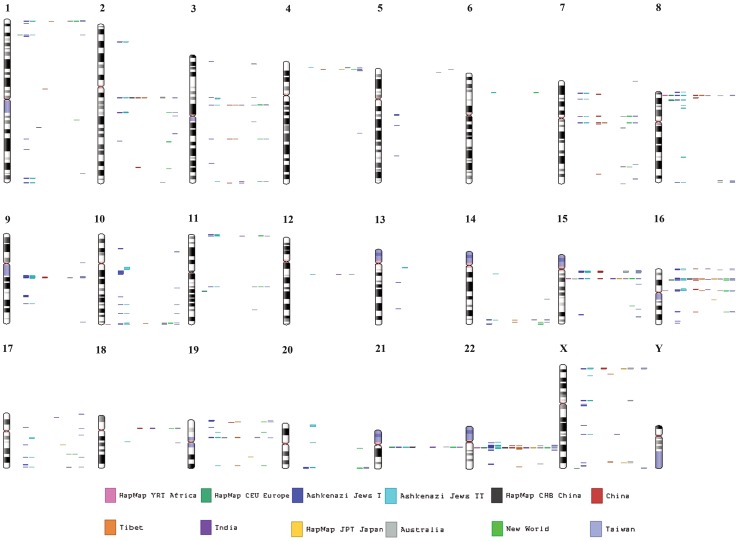
Map of miRNA genes in different chromosomes across populations. Each color coded vertical line represents a single population.

We identified a total of 44109 CNVs investigated from 1715 individuals across 12 populations. These CNVs contain 126190 genes consisting of 15185 singleton genes. Of these, 4007 miRNA-CNVs (∼9%) contained 6542 (∼5%) miRNA genes with a total of 333 (∼5%) singleton miRNA genes. These miRNA-CNVs were identified in 1438 (∼83.8%) individuals with a mean of 4.5 miRNA genes (Table 1) (Figure 1). miRNA-CNVs were found ranging from 3–11% across all populations with Taiwan, Ashkenazi Jews and Australia showing >8% while the remaining populations were found between 4–7% and YRI with only 3%. Duplication CNVs containing miRNA genes was significantly higher (87.6%) compared to deletion (12.4%). However, HapMap populations excluding YRI showed higher miRNA deletion CNVs than duplication CNVs. The duplication miRNA-CNVs were in the range of 81–91% with highest in Ashkenazi Jews (91.6%) and lowest in CHB (36.8%). The deletion miRNA-CNVs were found highest and lowest in CHB (63.1%) and Tibet (5.6%) respectively.

**Table 1:** Distribution of miRNA CNVs and genes across 12 populations.

				miRNA CNVs				miRNA Gene count			
Population	Total Individuals assessed	Individuals with miRNA CNVs	Total miRNA genes identified	Duplication (%)	Deletion (%)	Global Mean miRNA CNVs	Total Non- miRNA CNVs	Duplication (%)	Deletion (%)	Mean of total miRNA genes	Total Singleton genes identified
**HapMap-YRI-Africa**	90	25	6	57.14	42.85	0.69	9.74	57.14	42.85	0.69	0.8
**HapMap-CEU-Europe**	90	44	14	44.44	55.55	1.34	11.76	51.61	48.38	1.34	1.75
**Ashkenazi Jews I**	464	441	173	88.78	11.21	31.14	27.8	82.66	17.33	31.14	22.53
**Ashkenazi Jews II**	480	468	141	91.64	8.35	33.46	26.41	83.77	16.22	33.46	18.89
**HapMap-CHB-China**	44	14	30	36.84	63.15	0.47	9.7	20	80	0.47	1.34
**China**	155	93	60	80.68	19.3	4.39	18.64	33.33	66.66	4.39	3.91
**Tibet**	31	31	19	94.3	5.63	1.77	33.9	78.89	21.1	1.77	7.96
**India**	38	30	24	85.18	14.81	1.34	30.7	82.08	17.91	1.34	2.56
**HapMap-JPT-Japan**	45	20	121	38.46	61.53	0.64	10.73	42.85	57.14	0.64	3.1
**Australia**	53	51	47	89.3	10.69	4.66	43.03	88.81	11.18	4.66	16.19
**New World**	41	36	10	81.88	18.11	3.16	47.51	69.1	30.89	3.16	5.93
**Taiwan**	184	181	118	87.86	12.13	16.87	34.39	82.04	17.95	16.87	14.97

### Chromosome-wise distribution of miRNA-CNVs

Chromosome 22 showed the highest CNV burden on miRNA genes across all populations (Figure S1a). Chromosomes 15 and 16 further showed the mean burden at ∼9% and 11%, with Ashkenazi Jews and JPT showing heavy burden. There were few instances without miRNA-CNV presences in chromosome, for populations such as CHB and Tibet showing no CNV presences in chromosome 15, whereas, chromosome 16 showed miRNA-CNV presences for all populations. Chromosomes 13, 5 and 6 showed negligible presence of miRNA-CNVs for all populations, population specific miRNA-CNVs presence or absences were also observed (Figure S1a).

### miRNA Gene concentration in duplication and deletion regions

**Figure 2:**
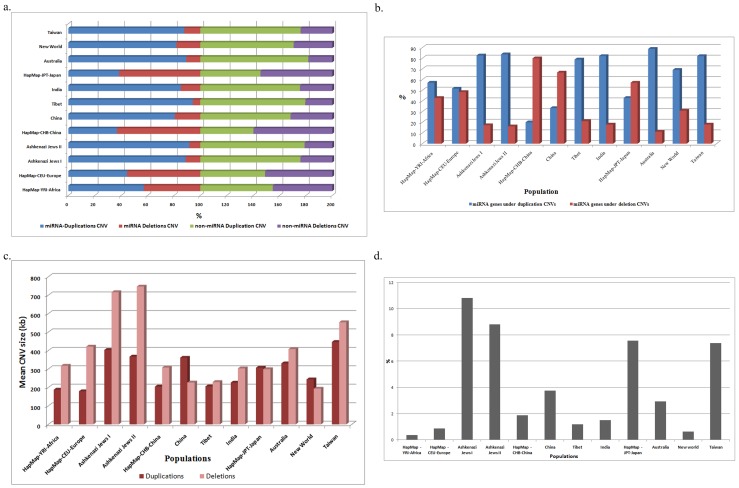
Distribution of miRNA CNVs and genes across 12 populations. a) Represents distribution of miRNA and non-miRNA CNV types b) represents burden of miRNA genes under duplication and deletion CNVs; c) represents mean duplication and deletion CNV size; d) represents the percent distribution of singleton miRNA gene.

miRNA genes were found overrepresented in duplication regions in eight populations, and deletion regions in three populations and near to equal ratios in only one population. The highest gene content under duplication CNVs was observed for Australia and Ashkenazi Jews, followed by India, Taiwan and Tibet (Table 1). The highest gene content under deletion CNVs was observed in Chinese populations (CHB-80%, China-66.6%) and JPT (57%) (Figure 2b). miRNA-CNV size across population revealed contrasting distribution compared to CNV occurrence showing significant contribution from sizes of deletion CNV than duplication. The highest deletion CNV size was observed for eight populations and equal ratios of miRNA gene were observed in both duplication and deletion regions only for JPT. Ashkenazi Jews and Taiwan populations showed heavy burden of CNV size on miRNA genes with a mean CNV size ranging from 400–700 kb, while the miRNA-CNVs of the remaining populations ranged anywhere between 200–400 kb (Figure 2c). While the former was due to both duplication as well as deletion miRNA-CNVs, the latter was burdened by only the deletion miRNA-CNVs.

Of the total miRNA singleton genes under CNVs Ashkenazi Jews, JPT and Taiwan showed the highest number (7–11%) while Africa and New World populations showed the lowest number (Figure 2d). First glances of total miRNA gene distribution across chromosomes revealed 22^nd^ chromosome to contain large number (30–60%) of miRNA genes under CNVs, however, singleton miRNA gene distribution across chromosomes revealed otherwise (<10%). This disparity was observed due to the consistent frequenting of CNVs in the regions bearing mir-650 gene across all populations and not due to multiple genes (Figure S1b). This type of disparity between the distributions of total genes and singleton miRNA genes was also observed across chromosomes within the populations (Figure S2a–b).

### Copy Number (CN) State of miRNA sub-genome

**Figure 3:**
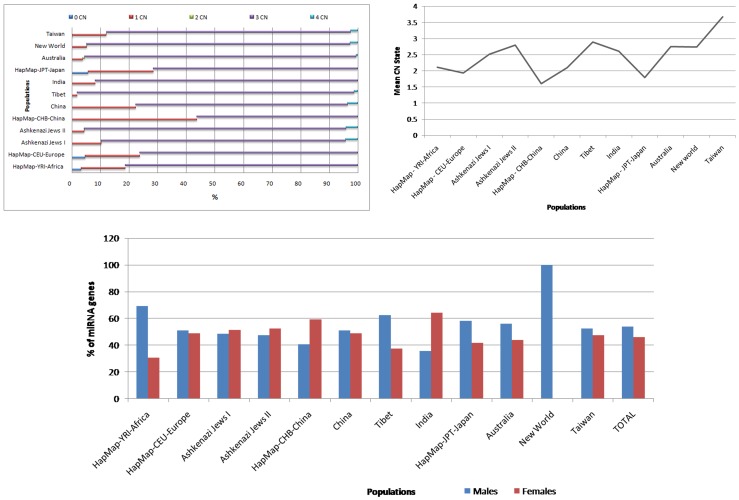
Copy Number State and Sex bias in miRNA sub-genome. a) Distribution of copy number states of the miRNA CNVs across 12 populations; b) represents the mean CN state across populations and c) represents the male-female distribution of miRNA genes under CNVs.

CN states for all duplication and deletion miRNA-CNVs were assessed based on the 0, 1, 2, 3 and 4 states, where the numerical value represents its corresponding allele presence in the genome. HapMap and China showed complete removal of both allelic segments (CN = 0) in the genome (18–45%) for some miRNA genes. Taiwan, Ashkenazi Jews, India, Australia and Tibet showed a further loss of one allele state in the miRNA gene bearing regions (<11%). Nearly all populations showed a high number of CN = 3 autosomal duplication miRNA- CNVs ranging from 58–80%. The homozygous duplication (CN = 4) state was seen across all populations distinctly, Ashkenazi Jews, China, Taiwan and New World showed the highest dual duplication CN state (3–5%) (Figure 3a). Inclusive analyses of all the 5 CN states showed increased losses of miRNA sub-genome in CHB, JPT, CEU, China, YRI and on the contrary Tibet, Ashkenazi Jews, Australia, New World, India and Taiwan showed significant overall gains, while CN state in the miRNA genes of sex chromosomes (CN = 2) was observed only for Australia. Mean CN state across all populations ranged from 1.6–3.7. The CN state of two populations (CHB and JPT) were <2 while the remaining populations varied between 2–3 (Figure 3b).

### Sex bias in miRNA-CNVs

miRNA-CNV gene presences were observed to be biased in male and female genomes in several populations (Figure 1c). Africa, Tibet, JPT and Australia showed males (58–60%) to be carrying more miRNA genes under CNVs than females (≤40%), while, CHB and India showed higher content in females (∼60%) compared to males (≤40%). The remaining populations showed balanced miRNA genes in both sexes, and New World population did not contain female samples, but show the largest miRNA gene content in males, whereas, Indian males showed the lowest miRNA gene content under CNVs (Figure 3c).

### miRNA-CNV Inheritance and Mutation rate

The miRNA-CNV rate analysis on trio families of YRI, CEU and India, revealed three clusters of miRNA-CNVs based on inheritance. CNVs with precise breakpoints inherited from one generation to the next bearing the exact size and present in the same location were regarded as ‘Inherited’ miRNA-CNVs. miRNA-CNVs which were not identified in the first generation but appeared in the second one with distinctly clear breakpoints non-overlapping with other CNVs were regarded as *De novo* miRNA-CNVs. The third cluster contained CNVs which showed atleast one breakpoint (either start point or end point) being precisely inherited, and the other non-inherited, but extended breakpoint was considered as *de novo*, collectively they were referred to as “Unknown”. Of the 3,103 CNV calls examined 1160 CNV breakpoints were identified in the probands. Of these, 175 were assigned as inherited and 668 were *de novo* CNVs The remaining 298 were the unusual ‘Unknown’ CNV events for which a single parental origin could not be assigned. 53 miRNA-CNVs from 1160 CNV breakpoints were further used to study the miRNA-CNV inheritance and *de novo* mutation rate. YRI showed 8% of the miRNA-CNVs being inherited, while India showed nominal rate of 4.7% and CEU did not contain any inherited miRNA-CNVs. However, *de novo* mutation rate was high for YRI and CEU while, the third cluster showed unequal rates with CEU being the highest (37.5%) followed by YRI (16.6%) and India (14.2%). The inherited miRNA-CNVs clustered above based on the point and type of origin showed contributions only from the maternal origin.

The investigation on miRNA-CNV hotspot rates is also critical in understanding the CNV mutation rate and genomic instability in the etiology of the miRNA regulated traits. The entire 4007 miRNA-CNV calls were used as an input to detect recurrent and unique copy number regions based on percent overlap. miRNA-CNV Hotspots were observed in chromosomes 22, 16, 15, and 3 whereas, chromosomes 13, 5 and 6 showed fewer hotspot CNVs. Unique and intermediate events were distinctively found across all chromosomes of the populations.

### Minor Allele Frequency (MAF) of miRNA genes

We identified 16 miRNA genes to be under tremendous burden of CNVs across all populations. These miRNA genes were found in varying frequency (1–15.3%) under several miRNA-CNV breakpoints, both within and across populations (Table 2). Though, both duplications and deletions were observed for these genes, however, duplications were more prevalent. These miRNA-CNV breakpoints were identified starting from nine populations upto six populations as we did not find any miRNA breakpoints shared between 12 to 10 populations. A total of 18 singleton breakpoints were found across populations distributed in chromosomes 22, 15, 16 and 3. The highest global frequency (15.3%) was observed for 14 miRNA-CNV breakpoints containing mir650 gene located in chromosome 22q11. The pair-wise clustering of shared miRNA genes in percent across all chromosomes and 12 populations is represented in the Circos image (Figure S3).

**Table 2:** Population-wise and global frequency of recurrent miRNA-CNVs shared across 12 populations.

Chr Location	Start Point	End Point	Genes	HapMap-YRI-Africa	HapMap-CEU-Europe	Ashkenazi Jews I	Ashkenazi Jews II	HapMap-CHB-China	China	Tibet	India	HapMap-JPT-Japan	Australia	New World	Taiwan	Total
**9 Populations**																
**22q11.22**	23040138	23255556	MIR650	4	-	3.4	0.42	-	2.15	6.45	3.33	5	1.96	-	2.21	2.02
**22q11.22**	23090867	23213354	MIR650	20	-	3.17	3.63	7.14	1.08	6.45	3.33	-	-	2.77	1.1	3.07
**8 Populations**																
**16p13.11**	14989851	15116245	MIR3180, MIR3179	-	-	1.13	1.07	-	5.37	3.23	-	5	1.96	2.77	2.21	1.6
**22q11.22**	23083891	23223233	MIR650	4	9.09	6.12	2.99	-	1.08	3.23	-	-	5.88	-	1.66	3.77
**22q11.22**	23099160	23223233	MIR650	4	4.55	-	1.06	-	1.08	-	3.33	5	1.96	-	2.21	1.16
**22q11.22**	23101771	23258994	MIR650	4	-	0.23	0.64	7.14	1.08	3.23	-	-	1.96	-	0.55	0.69
**22q11.22**	23111958	23251602	MIR650	-	2.27	6.34	9.4	7.14	3.23	6.45	3.33	-	-	-	2.21	5.86
**7 Populations**																
**22q11.22**	22943462	23266118	MIR650	-	-	4.08	5.55	-	2.15	-	3.33	-	15.69	-	1.1	3.97
**22q11.22**	22997799	23275341	MIR650	-	-	9.98	10.04	-	6.45	9.68	3.33	-	-	25	38	10.32
**22q11.22**	23046123	23223233	MIR650	4	2.27	3.62	7.47	-	7.52	3.23	-	-	-	-	1.66	4.46
**15q14**	34695310	34830932	MIR1233-2	4	-	0.23	0.64	-	1.08	-	3.33	-	-	2.77	0.55	0.63
**6 Populations**																
**22q11.22**	23051908	23223233	MIR650	-	-	2.49	5.98	-	5.38	-	-	-	15.69	2.77	1.1	3.83
**22q11.22**	22871748	23251602	MIR650	-	-	0.91	0.85	-	1.08	3.23	-	-	1.96	-	0.55	0.84
**22q11.22**	22962462	23223233	MIR650	-	-	3.85	2.77	-	2.15	16.13	-	-	7.84	-	7.73	3.83
**22q11.22**	23078678	23268562	MIR650	-	-	0.45	4.49	-	5.38	3.23	10	-	-	-	3.31	2.65
**22q11.22**	23112739	23223233	MIR650	-	-	4.53	0.85	-	1.08	6.45	-	-	11.76	-	0.55	2.37
**15q14**	34727610	34857998	MIR1233-2	4	2.27	0.23	0.21	-	-	-	-	5	-	-	0.55	0.42
**3p12.3**	75500752	75682306	MIR1324	-	-	0.68	0.21	-	1.08	6.45	-	-	-	16.66	6.07	1.67

### CNV-miRNA Pathway

**Figure 4:**
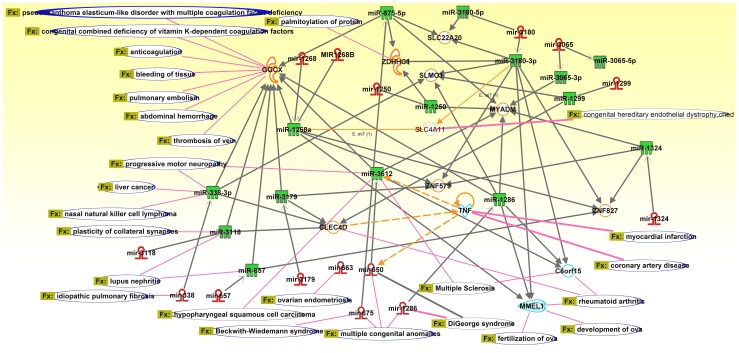
CNV miRNA pathway showing the CNV burden of miRNA genes and its interaction with target proteins indicating the involvement in biological functions and diseases.

The miRNA genes which were found to be under severe CNV burden are mir650, mir3179, mir3180, mir1324, and mir1233-2 along with 12 other miRNA genes. In the current study we made use of Ingenuity Pathway Analysis (IPA) to identify the functional processes of miRNA genes under the CNV load and to further assess the possible phenotypic alterations in the population. IPA revealed 23 diseases and disorders for various organ systems, and these further contained 26 function-specific disease annotations including the direct regulators of miRNAs and their target genes. We identified a total of >112 proteins being regulated directly or indirectly by >15 pre-miRNA molecules and ∼15 mature miRNA molecules. Several of these molecules were found to be synergistic in both the regulation of various mRNA targets and in causing diseases. We found a majority of the miRNAs to be regulating several transcripts and a few proteins regulating specific miRNAs. For instance, Tumor Necrosis Factor (TNF) regulates both mir650 pre-miRNA and mature miRNA molecules. This interlink of mir650 (pre-miRNA), TNF and mir3612 (mature miRNA of mir650) were a major hub consisting of interlinking proteins and more miRNA molecules further regulating the downstream and upstream processes (Figure 4). Disruptions in these processes were found to affect multiple processes resulting in severe disease phenotypes, both common and rare. GGCX is another hub which is regulated by vast amount of miRNA genes and has functions related to immune system development. There were several major and minor hubs in this CNV-miRNA pathway, most of them were interlinked increasing the complexity of the regulation.

## Discussion

Identification of the miRNA-CNVs across diverse populations helps understand the organization, distribution pattern of miRNA-CNVs, evolutionary dynamics of the miRNA sub-genome, and account for differences in the expression of genes. There have been only few miRNA-CNV investigations on the CNVs obtained through bioinformatic identification from several databases [12] and HapMap populations [9]; however, there have not been many, which comparatively include populations across all continents to study notable variations on the genome, particularly on genes using different ethnic backgrounds. The current study represents the first drafts of population-specific miRNA-CNVs maps as well as a cross-populational map. Here we present a comprehensive global miRNA-CNV spectrum by identifying 333 singleton miRNA genes from total of 6542 miRNA genes from 4007 miRNA-CNVs across 12 populations using Affymetrix high resolution arrays. The CNVs identified in this study are highly consistent, because of the higher stringency adopted in both the selection and validation of CNVs using multiple algorithms. Although these samples are well characterized, no medical information (except for HapMap) was obtained. The miRNA genes and CNVs were co-active and conferred tremendous burden in the regulation of the coding genome. Genetic diversity in humans affects both disease and normal phenotypic variation. Presence of CNVs alters the transcriptional and translational levels of overlapping or nearby genes by disrupting the coding structure or by altering gene dosage thereby conferring differential susceptibility to complex diseases [28]–[34] and presence of miRNA-CNVs further adds to the complexities on the regulation and coding structure of the genome.

We detected gradual increase in the miRNA-CNV counts from the Old World populations towards the New World populations (Bolivians and Totanacs) indicating selective pressure of CNV occurrences in New World populations contributing to increased genetic diversity. miRNA-CNVs can considerably alter their dosage, which would then affect the expression levels of the target genes [3], [28], [35]–[38]. We identified both miRNA and non-miRNA duplication CNVs to be higher than deletion CNVs in several populations, while only few populations showed increased deletions. The prevalence of higher miRNA-duplication CNVs might be due to the lesser damage it offers by protecting against deletions [38]. Though, duplication CNVs have been associated in many diseases, most of them are regarded due to partial duplication disruptions of the coding gene. No consistent pattern was identified for the miRNA duplication CNV distribution across ethnicities since extremes of both CNV types were found elevated in ethnic groups residing within the continental boundaries. Wu et al. [9] observed CNVs in the miRNA regions of the HapMap populations, and their data significantly overlaps with our findings of CNVs in HapMap populations which indicates that the CNVs identified in this study are highly consistent. The observations of Wu et al. [9] and Marcinkowska et al. [12] greatly differed with our findings and were not comparative across all study populations.

### Chromosome-wise distribution of miRNA-CNVs

Chromosome 22 harbors high concentration of miRNA-CNVs followed by chromosomes 15 and 16. The reason behind such chromosomal selection for miRNA-CNVs to frequent is largely speculative. These three chromosomes contain three miRNA genes, namely, mir650 in chromosome 22, mir1233-2 in chromosome 15 and mir3179/80 in chromosome 16. These genes were found largely under CNVs across all populations, however, population specific miRNA-CNV presences were also observed for India, Tibet and New World. Several miRNA-CNVs have been implicated in pathways, phenotypes and diseases [15], [39]–[42] while various miRNA-CNVs are found to be either deleted or duplicated in diverse types of cancers [43]–[48]. The observed varied dominance of chromosomal miRNA-CNVs may help in understanding the molecular basis of human phenotypic diversity.

We observed higher duplication events than deletions in several studies [9], [12]; similarly the genes under these CNVs also tend to be overrepresented in duplication CNVs compared to deletion CNVs. New World showed higher gene content in miRNA duplication CNV events, while gene overrepresentation in deletion CNVs were limited to only China and Japan. These indicate the genetic relationships and diversity between and within populations and thus we propose that miRNA-CNVs are inherent variants and should be considered as probable candidates while performing genotype-phenotype association studies.

miRNA-CNV size and miRNA-CNV count distribution deviated significantly across population and revealed contrasting contributions towards the burden of CNVs. Though we found duplication miRNA-CNVs to be overrepresented than deletion CNVs, however, further analysis on miRNA-CNV size, revealed larger size deletions to be encompassing miRNA genes compared to duplication CNVs while only few populations were found with high duplication sized miRNA-CNVs. This probably indicates that, CNV type, count and size factors are independent of each other and should be collectively analyzed to understand the complexities of miRNA in populations.

Ashkenazi Jews, JPT and Taiwan contained the highest number of singleton miRNA genes under CNVs, than the remaining populations. The inconsistency in total miRNA genes distribution against singleton miRNA gene counts across chromosomes was observed due to the regular frequenting of CNVs in regions bearing one gene across many individuals in all populations and was not due to the presence of multiple miRNA genes and this pattern was observed across many chromosomes within the populations.

### Sex bias

Gender plays a pivotal role in the human genetic identity and is also manifested in many genetic disorders. miRNA-CNV presence showed sex bias in several populations, largely dependent on ethnicity. There was a significant difference in miRNA gene distribution under CNVs in both male and female genome. These findings imply gender based phenotypic differences. Many human genetic phenotypes, including those related to olfaction, developmental delay and intellectual disabilities have shown similar sex bias along with recombination rates [49]–[50].

It was necessary to evaluate the zygosity nature of all the identified miRNA-CNVs to accurately assess the genome changes in order to correlate with duplication and deletion events. HapMap and China showed significant losses in the miRNA sub-genome with increased 0 and 1 deletion CN states, while remaining populations showed higher duplication CN state of 3 and 4 indicating homozygous gain of parts of miRNA sub-genome. Australia exhibited burden of miRNA-CNVs on sex chromosomes compared to any other population, which indicates the phenotypic differences of the Australian population can largely be attributed to the miRNA genes present on sex chromosomes. No distinct pattern could be derived from the observed CN states of all the populations, since they all exhibited differences even with shared ethnicities. Comparison of the 5 CN states of the non-miRNA CNVs with the CN states of miRNA-CNVs revealed striking similarities indicating that the miRNA sub-genome is under the force of CNV dynamics of the genomes and that the miRNA sub-genome does not seem to show any independent force acting on it.

Based on the inheritance status, YRI showed a fold higher inheritance rate compared to India, whereas CEU did not show any inheritance of miRNA-CNVs. The rate of *de novo* CNV occurrences were similar, while “Unknown” cluster showed unequal rates with YRI being the highest, followed by CEU and India. The reasoning and the mechanism on the origin of “Unknown” CNVs is yet to be clearly understood. All the miRNA-CNVs clustered based on the point and type of origin showed biased contributions of miRNA-CNVs from mother compared to father. Though, earlier studies have reported diverse CNV transmission and *de novo* event rates in probands with several neurodevelopmental phenotypes and monozygotic twin studies [51]–[54], however, no such inheritance rate on the miRNA-CNVs has been performed before. miRNA-CNV frequency bias was observed on the CNV transmissions from maternal genome only, showing major contributions from deletion CNVs than duplications. We believe the existence of such biased transmissions greatly signifies the role of parental transmission in gene regulation and counters some distinct phenotypic features observed in previous studies [55]–[56] and in certain gender-specific disease manifestations.

### Hotspot Detection

Hotspot analysis was performed using HD-CNV to identify the hotspots and unique CNVs between samples of the same and other populations and to also correlate their abeyant effects on a wide variety of regulatory pathways. HD-CNV program was used on 3958 miRNA-CNV events revealing hotpot, rare and intermediate miRNA-CNV regions between samples of the same and other populations. Unique CNV events were considerably higher compared to hotspots and intermediate events, and almost all events were found overlapping with atleast one other event. These events are very commonly recurring events that have similar boundaries and not much distinction can be observed in the groups. Chromosomes 22, 16, 15 and 3 shows highly conserved patterns, as there are large groupings of overlapping CNV calls, and very few individual calls. The locus-specific mutation rates for CNV have been observed to be ∼100 to 10,000 times higher than those for nucleotide substitution rates, which not only highlights the instability of CNV regions but also suggests large variation in CNV mutation rate [57]. The data from the 1000 Genomes Project suggest that the distribution of CNVs in the genome is biased towards multiple hotspots including segmental duplications and away from genes encoding protein complexes and other dosage sensitive genes [58]. Contrary to this, our data suggests miRNA-CNV distribution in regions of the genome bearing miRNA genes regulating protein complexes and dosage sensitive genes. Hotspot analysis further elucidated the fragility of the miRNA sub-genome which contains recurrent CNV regions bearing large concentration of repeats in the flanking sequences. Some of these recurrent CNV regions are the probable reason behind several miRNA genes showing high copy numbers across populations.

Around 16 singleton miRNA-CNVs from a total of 4007 miRNA-CNVs are identified across chromosomes in all populations at varying frequencies. Many of the HapMap miRNA-CNVs identified here are found in previous investigations [59], this hence validates the ability of our arrays to detect reliable variations. Our analysis of different populations allowed an assessment of population-specific miRNA-CNVs. These CNVs across multiple ethnic populations suggest that these structural rearrangements confer phenotypic diversity to a great extent.

### miRNA-CNVs Functional and Disease Pathway

We computed a network for the individuals containing CNVs in both miRNA genes and in their protein-coding gene targets to quantify burden and to determine variability within and between populations. We selected the 16 miRNA genes that were heavily burdened by CNVs and established a regulatory network involving miRNAs, their targets and the target's interacting partners. Assessing relatedness between the miRNA molecules revealed mechanisms that influenced disease susceptibility and phenotype expression (Table 3).

**Table 3:** Functional and disease association annotation of the miRNA genes under the CNV burden.

Category	Functions Annotation	p-Value	Molecules
Cardiovascular Disease	DiGeorge syndrome	2.52E-05	mir-1286, mir-650
Congenital Heart Anomaly	DiGeorge syndrome	2.52E-05	mir-1286, mir-650
Developmental Disorder	DiGeorge syndrome	2.52E-05	mir-1286, mir-650
Hereditary Disorder	DiGeorge syndrome	2.52E-05	mir-1286, mir-650
Inflammatory Disease	Lupus nephritis	6.85E-05	miR-3118, miR-657, TNF
Hereditary Disorder	Harboyan syndrome	6.99E-04	SLC4A11
Ophthalmic Disease	Harboyan syndrome	6.99E-04	SLC4A11
Developmental Disorder	Congenital hereditary endothelial dystrophy, ched 2	6.99E-04	SLC4A11
Hereditary Disorder	Congenital hereditary endothelial dystrophy, ched 2	6.99E-04	SLC4A11
Ophthalmic Disease	Congenital hereditary endothelial dystrophy, ched 2	6.99E-04	SLC4A11
Inflammatory Disease	Cytomegalovirus colitis	6.99E-04	TNF
Gastrointestinal Disease	Cytomegalovirus colitis	6.99E-04	TNF
Infectious Disease	Cytomegalovirus colitis	6.99E-04	TNF
Organismal Injury and Abnormalities	Deformation of tail	6.99E-04	TNF
Ophthalmic Disease	Fuchs endothelial corneal dystrophy type 4	6.99E-04	SLC4A11
Skeletal and Muscular Disorders	Inflammation of cremaster muscle	6.99E-04	TNF
Connective Tissue Disorders	Insulin resistance of white adipocytes	6.99E-04	TNF
Endocrine System Disorders	Insulin resistance of white adipocytes	6.99E-04	TNF
Metabolic Disease	Insulin resistance of white adipocytes	6.99E-04	TNF
Hereditary Disorder	Pseudoxanthoma elasticum-like disorder with multiple coagulation factor deficiency	6.99E-04	GGCX
Connective Tissue Disorders	Pseudoxanthoma elasticum-like disorder with multiple coagulation factor deficiency	6.99E-04	GGCX
Cancer	Transformation of dendritic precursor cells	6.99E-04	TNF
Inflammatory Disease	Multiple Sclerosis	9.20E-04	C6orf15, miR-3612, TNF
Skeletal and Muscular Disorders	Multiple Sclerosis	9.20E-04	C6orf15, miR-3612, TNF
Neurological Disease	Multiple Sclerosis	9.20E-04	C6orf15, miR-3612, TNF
Neurological Disease	Progressive motor neuropathy	1.08E-03	C6orf15, miR-338-3p, miR-3612, TNF
Developmental Disorder	Congenital combined deficiency of vitamin K-dependent coagulation factors	1.40E-03	GGCX
Hematological Disease	Congenital combined deficiency of vitamin K-dependent coagulation factors	1.40E-03	GGCX
Immunological Disease	Delay in formation of granuloma	1.40E-03	TNF
Liver Damage	Hepatotoxicity of hepatocytes	1.40E-03	TNF
Dermatological Diseases and Conditions	Non-melanoma skin cancer	2.10E-03	TNF
Hepatic System Disease	Hepatitis subtype infantile hepatitis syndrome	2.79E-03	TNF
Liver Inflammation/Hepatitis	Hepatitis subtype infantile hepatitis syndrome	2.79E-03	TNF
Psychological Disorders	Scrapie of lymph node	2.79E-03	TNF
Drug Metabolism	Stimulation of hyaluronic acid	2.79E-03	TNF
Renal and Urological Disease	Unilateral ureteral obstruction	2.79E-03	TNF
Skeletal and Muscular Disorders	Wasting of skeletal muscle	2.79E-03	TNF
Nutritional Disease	Wasting of skeletal muscle	2.79E-03	TNF
Kidney Failure	Acute renal failure	7.09E-02	TNF
Liver Fibrosis	Fibrosis of liver	1.14E-01	TNF
Liver Cirrhosis	Cirrhosis	1.53E-01	TNF
Heart Failure	Heart failure	2.29E-01	TNF
Hepatocellular Carcinoma	Hepatocellular carcinoma	3.36E-01	TNF

*mir650* plays a key role in the manifestation of several cancers such as chronic lymphocytic leukemia (CLL) [60], colorectal [61], gastric cancer [62] and hepatocellular carcinoma (HCC) [63], furthermore, studies have also implicated dysregulation of mir650 in patients of diabetic ischemic heart failure [64] and multiple sclerosis. mir650 is known to be directly regulated by TNF, which are a group of cytokines which cause apoptosis. Upregulation of *mir650* down-regulates processes of ING4 favoring HCC tumorigenesis, while it targets ING4, cyclin dependent kinase 1 (CDK1) and early B-cell factor 3 (EBF3) molecules towards CLL prognosis.

mir3179/80, mir1324 and mir1268a are all found regulating ZNF579 target, while mir3180, mir3612 and mir1286 also regulate C6orf15, whereas TNF regulates mir3612. Overexpression of mir3179 has been implicated along with slightly altered TNF and IL-6 levels in Tuberculosis (TB) group compared to controls [65]. mir1286 down regulates C6orf15 and MMEL1; and alteration at these processes favor rheumatoid arthritis, and development and fertilization of ova. mir3179 and vast amount of other miRNA molecules such as mir338, mir3118, mir657, mir1268 and mir650 down regulates GGCX, and sometimes these down-regulators are regulated by other miRNA molecules such as mir650 which is master regulator controlling the GGCX downstream pathway, which also has been implicated in several disease processes such as pulmonary embolism, thrombosis of vein, abdominal hemorrhage and bleeding of tissue. mir675 is shown to up-regulate the essential cartilage matrix component COL2A1 essential in cartilage regeneration [66], while both H19 and mir675 are found to be upregulated in human colon cancer and primary human colorectal cancers [67]. These miRNA and target pathways are altered by disrupting or amplifying certain linear pathways by the occurrences of miRNA-CNVs influencing gene expression through different mechanisms such as, position effect, deletion or duplication of regulatory elements, and through variability of dosage. CNVs overlapping different parts of miRNA genes such as whole gene amplification increase the dosage; on the contrary, partial gene duplications and deletions can increase or decrease the dosage of miRNAs. This complex regulatory network consisting of CNV-miRNAs tend to target a higher average number of target genes and favors to synergistically regulate the same genes.

### Genotypic complexities provided by miRNA-CNV genes and its targets' network

**Figure 5:**
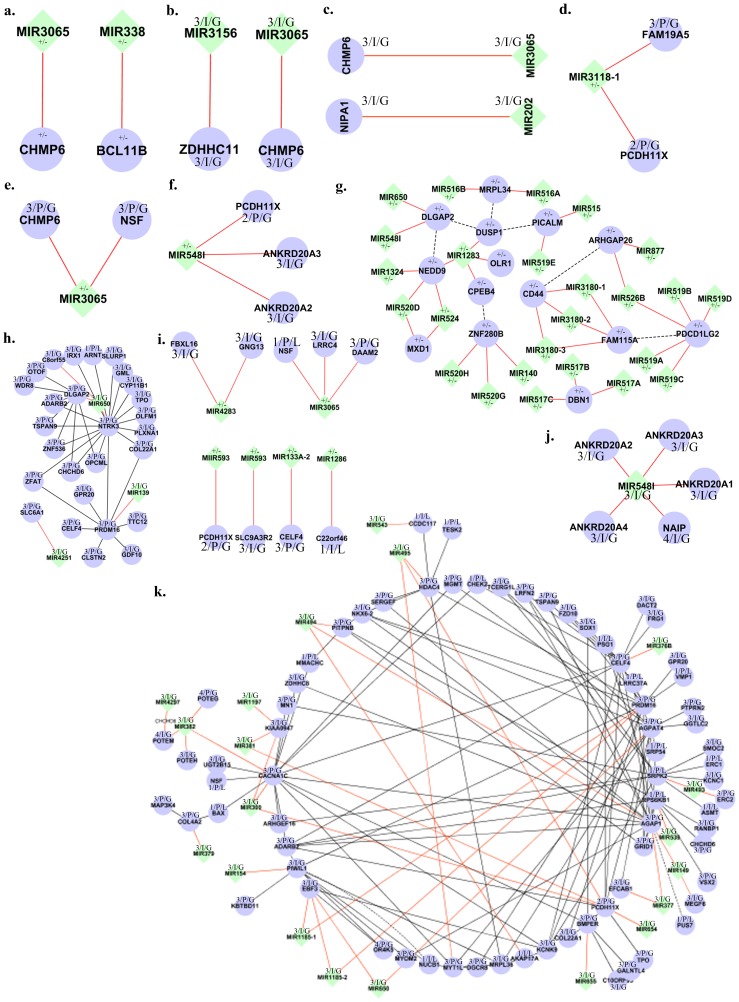
Complexities of miRNA CNV effect on genes. Representation of the different levels of complexities (a–h) present in the genome as a result of CNV influence on the miRNA genes, their targets and protein interactors. The ‘+’ and ‘−’ sign beside each gene indicates the CNV type of duplication and deletion, “I” and “P” indicates the overlapping status of CNVs and the numerical indicate the CN state.

A number of miRNA genes and its target partners are found to be under the burden of CNVs in individuals from New World, Australia and Tibet. Six miRNA genes and six targets are found disrupted by CNVs from an individual from New World, whereas, the individual from Australia showed disruption of several miRNA such as mir519b, mir526b, mir519d, mir519a, and mir519c and its target gene *PDCD1LG2*, furthermore mir526b and mir877 are disrupted along with its target *ARHGAP26*. In an individual from Tibet, mir517b, mir517a, and mir517c along with its target gene DBN1 are under the CNVs, further, mir3180-1, mir3180-2, and mir3180-3 and its target genes CD44 and FAM115A in the same individual are disrupted. A total of >100 miRNA genes and >500 target partners are identified in individuals from different populations to be under the direct burden of both duplication and deletion CNVs (Figure 5).

Variations bearing miRNAs cause diseases because of their failure to silence their target genes and since miRNAs have multiple targets, it is expected that they have a collective action over cell functionalities. To characterize the efficacy of miRNA-CNVs on regulation, we have identified several mechanisms for selected miRNAs to determine potential functional effect. These mechanisms are represented on a miRNA-CNV, miRNA-Target and, Target-interacting proteins network which helps in the better understanding of complexities of the CNVs in miRNA gene regions. Studies on the implications of CNVs on disease genes have been extensively performed; however, the layers of complexities remain unidentified. Although, there have been numerous investigations to find the influence of CNVs on gene expression phenotypes, however, only a few of them have effectively been able to associate them with diseases [68], while most part of it remains to be explored to understand phenotypic differences.

In the present investigation, several layers of complexities created due to the presence of multiple CNVs across many miRNA gene regions, which regulate gene targets within a genome, are extensively studied to unravel the complex mechanism of miRNA-CNVs towards the phenotypic diversity. This was performed by identifying the miRNA genes and their targets disrupted by the presence of CNVs. The following CNV-complexities were identified in the genomes of individuals: (i) CNVs disrupted the coding structure of ∼5 to >25 miRNA genes either by duplications or deletion of the entire or partial genes, (ii) The presences of CNVs in the genomic regions of the target genes of the above identified miRNA in the same individual, (iii) other miRNA genes under the CNVs which share similar target genes, (iv) CNVs truncating the coding structure of the immediate interacting protein partners of the miRNA targets as well as the miRNA genes disrupted through CNVs (Figure S4). For instance, CNVs were found to be disrupting the miRNA genes mir1324, mir1283, and mir520d in an individual, some of the miRNA targets which are shared between these disrupted miRNA genes were also found to contain a common miRNA target ‘NEDD9’ which was also under the influence of CNV. mir1283 down-regulates targets OLR1, CPEB4, and DUSP1, which are all under the influence of CNVs, further mir520d and mir524 also regulate MXD1. DUSP1 and NEDD9 are predicted to physically interact through DLGAP2 intermediary. Instances such as these were found in the genomes of many individuals. The layers of CNV complexities amplify with the increase of CNVs in either the target genes or the regulating genes.

This complex relationship of CNVs on miRNA-genes and its target partners unravels the complexities involved in gene function and phenotypic expression. We propose four different types of mechanisms which create the complexity in gene regulation indicating that multiple miRNA-CNVs, targets and their protein partners are potential functional variants. Therefore these proposed mechanisms should be considered as potential candidates while performing genotype-phenotype association and gene regulation studies. This complex relationship of CNVs on miRNA genes and their target partners unravels the many complexities involved in transcriptional regulation and phenotype expression.

## Supporting Information

Figure S1 **Chromosome-wise distribution of CNV burden across 12 populations.** a) Represents total percent of miRNA CNVs and b) represents the percent distribution of CNV size. Each cluster consists of 12 bars, each bar indicating a specific population.
(TIF)

Figure S2 **Chromosome-wise distribution of miRNA genes (in percent) across 12 populations.** (a) represents the total 6542 miRNA gene distribution across chromosomes and populations (b) is the 333 singleton miRNA gene distribution across chromosomes and populations. Each cluster consists of 12 bars and each bar indicates a specific population.
(TIF)

Figure S3 **miRNA CNV-gene map.** The outermost to innermost tracks represent the pair-wise clustering of total miRNA genes shared across all chromosomes and populations in this Circos image.
(TIF)

Figure S4 **The interaction complexity of miRNA CNV in function and/or disease in an individual.** miRNA genes interact with their specific target resulting in the disease and/or specific phenotype. CNV influencing the miRNA genes and their targets in the same individual may disrupt or alter the normal mode of interactions. Further, the CNV influence on protein interactors of the miRNA targets adding to the level of interaction complexity in the individual.
(TIF)
