# Supplementary figures and images for: Copy Number Variations Burden on miRNA Genes Reveals Layers of Complexities Involved in the Regulation of Pathways and Phenotypic Expression
Source: PLoS One. 2014 Feb 28;9(2):e90391. doi: 10.1371/journal.pone.0090391 (PMC3938728; doi:10.1371/journal.pone.0090391)

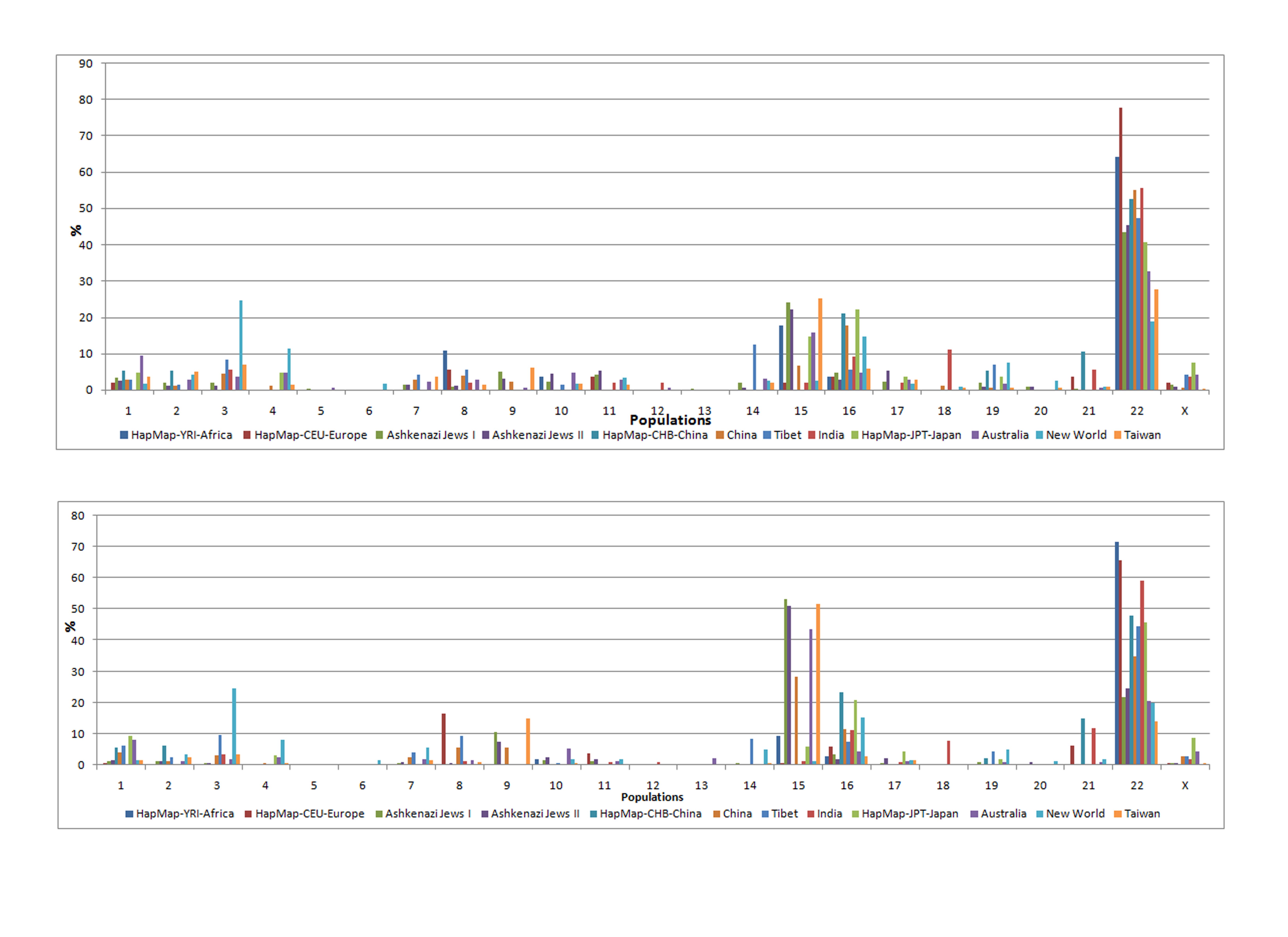

Supplement: Figure S1 — Chromosome-wise distribution of CNV burden across 12 populations. a) Represents total percent of miRNA CNVs and b) represents the percent distribution of CNV size. Each cluster consists of 12 bars, each bar indicating a specific population. (TIF) [file pone.0090391.s001.tif]

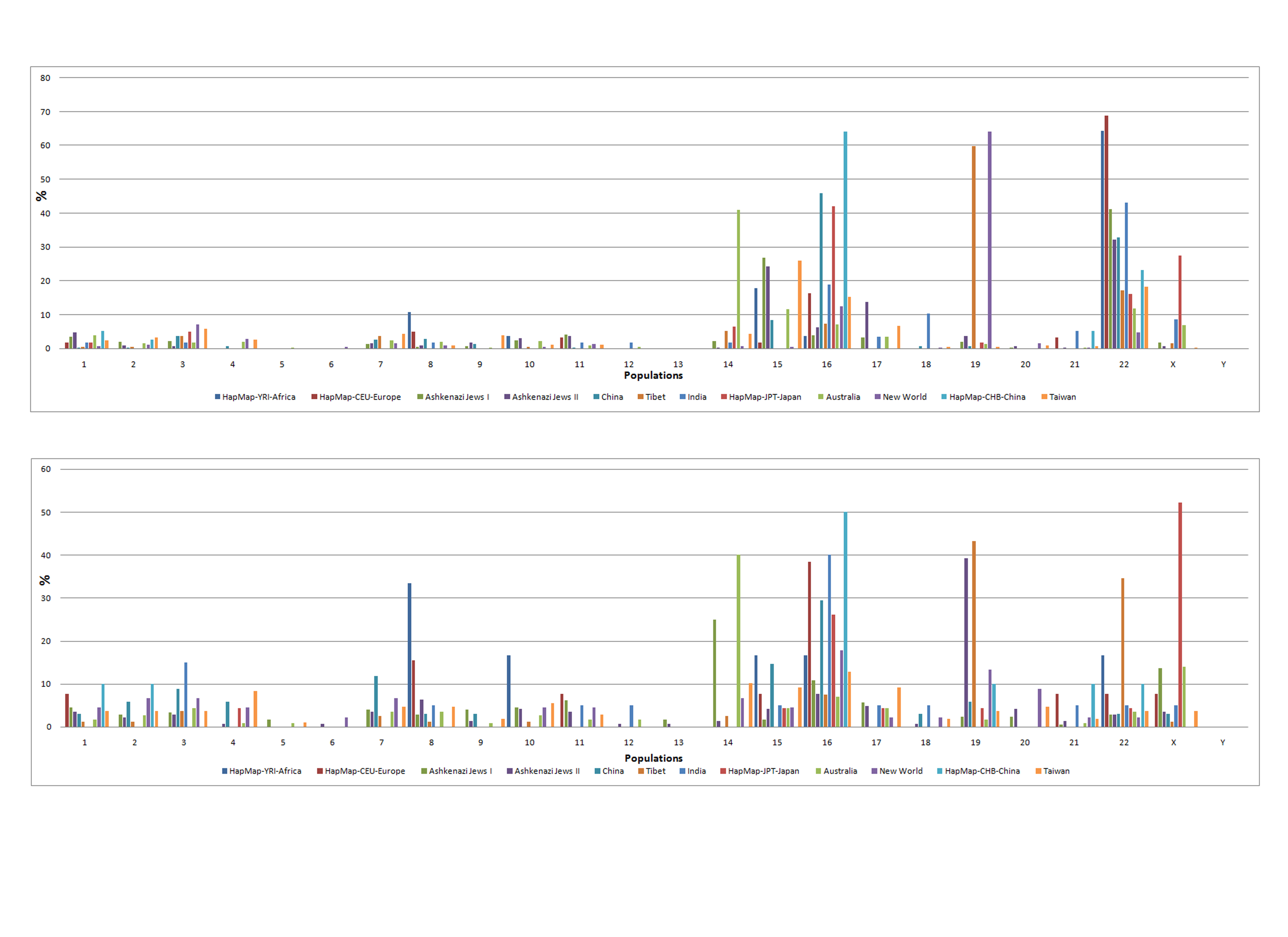

Supplement: Figure S2 — Chromosome-wise distribution of miRNA genes (in percent) across 12 populations. (a) represents the total 6542 miRNA gene distribution across chromosomes and populations (b) is the 333 singleton miRNA gene distribution across chromosomes and populations. Each cluster consists of 12 bars and each bar indicates a specific population. (TIF) [file pone.0090391.s002.tif]

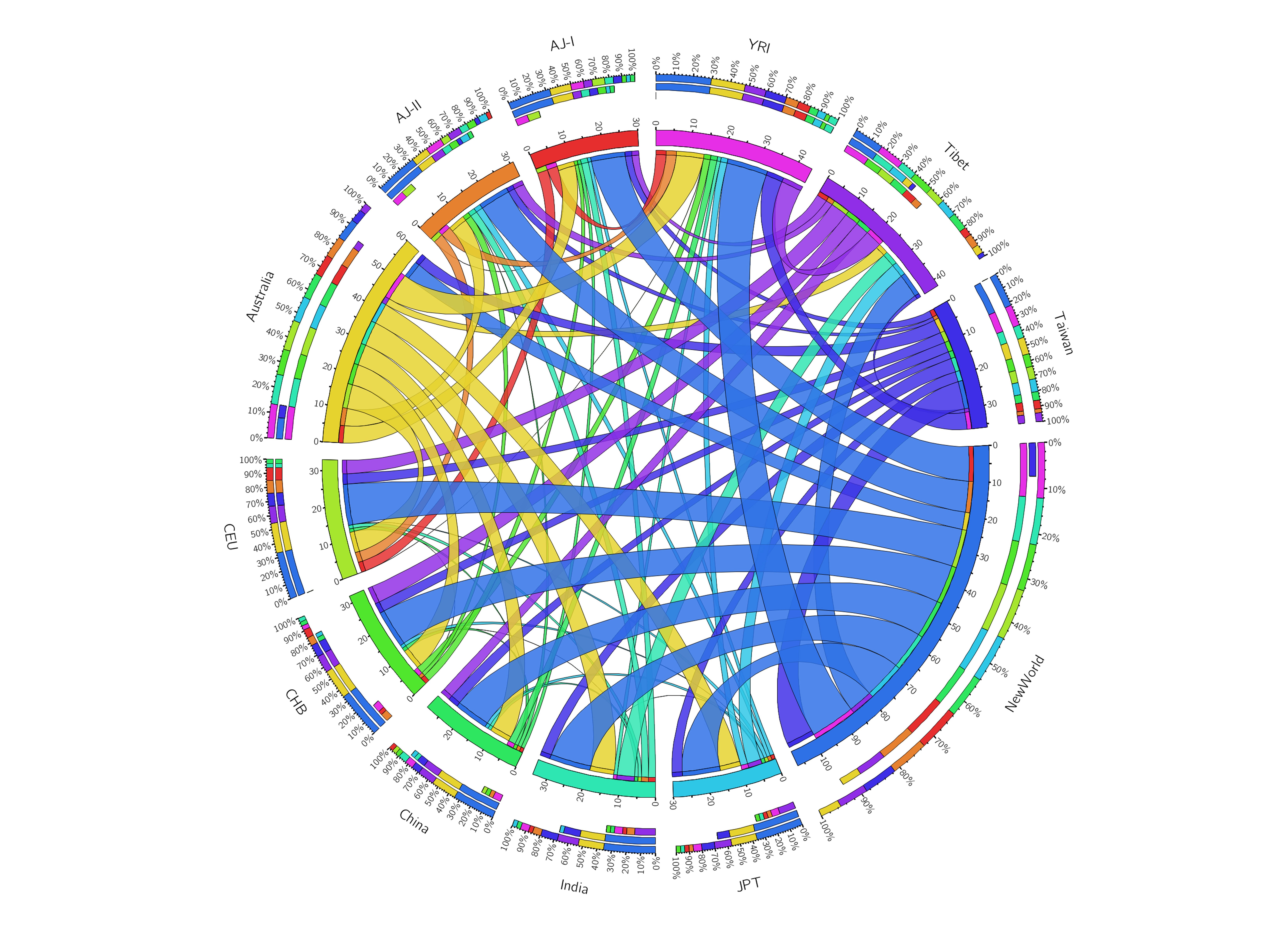

Supplement: Figure S3 — miRNA CNV-gene map. The outermost to innermost tracks represent the pair-wise clustering of total miRNA genes shared across all chromosomes and populations in this Circos image. (TIF) [file pone.0090391.s003.tif]

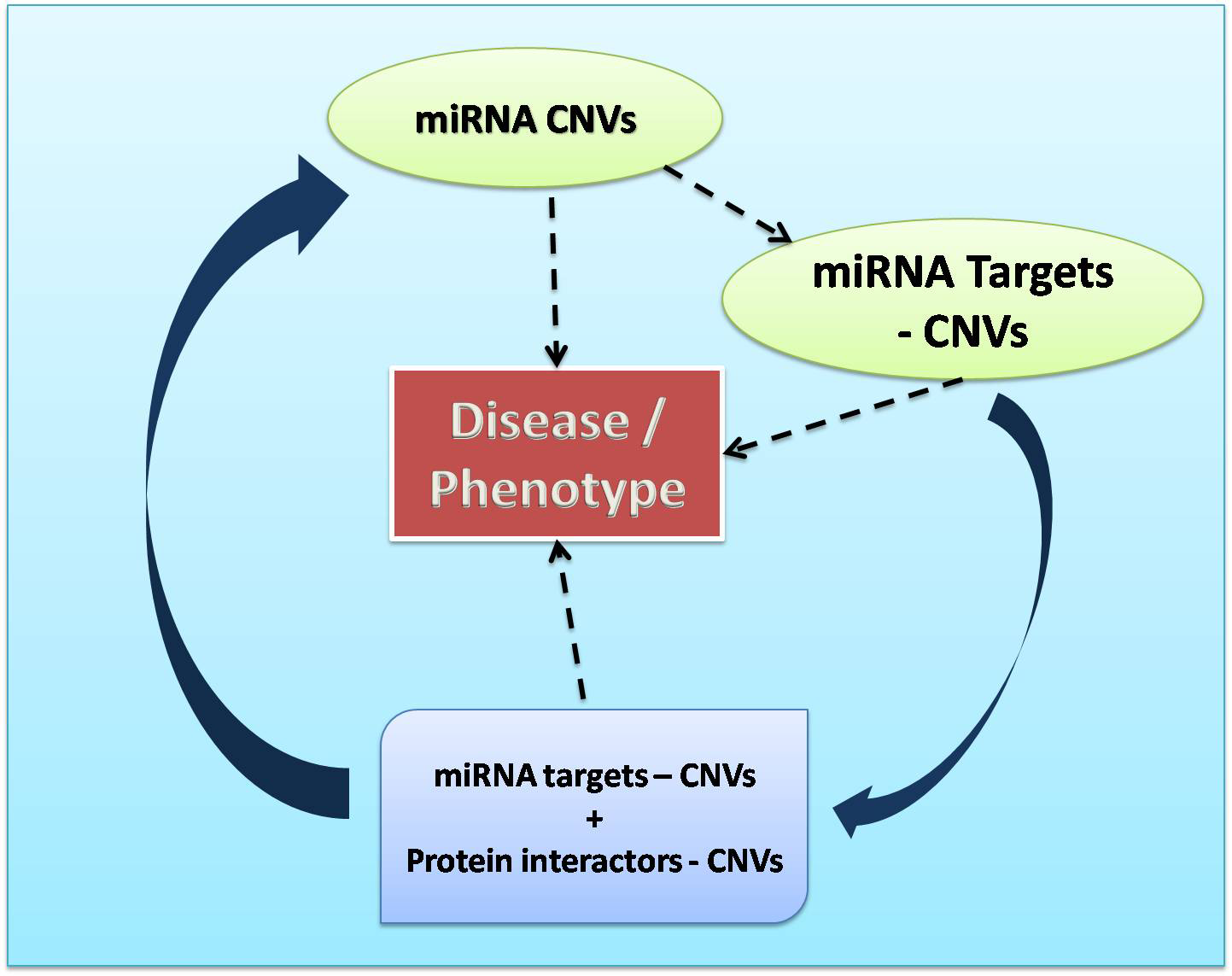

Supplement: Figure S4 — The interaction complexity of miRNA CNV in function and/or disease in an individual. miRNA genes interact with their specific target resulting in the disease and/or specific phenotype. CNV influencing the miRNA genes and their targets in the same individual may disrupt or alter the normal mode of interactions. Further, the CNV influence on protein interactors of the miRNA targets adding to the level of interaction complexity in the individual. (TIF) [file pone.0090391.s004.tif]
